# Back to Basics: A Clinical Diagnosis of Behavioural Variant Frontotemporal Dementia (bvFTD)

**DOI:** 10.1192/bjo.2026.11777

**Published:** 2026-06-30

**Authors:** Evangelos Galatis, Rebecca Chubb, George El-Nimr

**Affiliations:** North Staffordshire Combined Healthcare NHS Trust, Stoke-on-Trent, United Kingdom

## Abstract

**Aims:**

Behavioural variant frontotemporal dementia (bvFTD) is characterised by early and progressive changes in behaviour, personality and executive functioning. Neuroimaging and biomarkers can support diagnosis, these investigations may be normal in the early stages, increasing diagnostic uncertainty and potential misattribution to primary psychiatric illness. In such cases, a comprehensive neuropsychiatric assessment and collateral history remains key to diagnosis.

**Methods:**

A woman in her 50s was referred to neuropsychiatry with a 3-year history of disinhibition, emotional blunting, aggression, repetitive behaviours, and decline in social functioning. Assessment included neuropsychological testing (ACE-III and RBANS Update), brain MRI, FDG-PET, DAT scan, EEG, and CSF analysis (including autoimmune and infectious panels). Genetic testing was also undertaken. The case was reviewed by a dementia Neuroradiology multidisciplinary team and nuclear medicine consultant.

**Results:**

Despite clear and progressive behavioural and functional deterioration, structural and functional neuroimaging, EEG, and CSF investigations were initially unremarkable. Neuropsychological testing demonstrated multi-domain cognitive impairment, with particularly marked deficits in visuospatial ability and memory (≤1st percentile on RBANS). Behavioural features included public disinhibition, rigidity, aggression, loss of empathy, and impaired insight. No primary psychiatric disorder, delirium, or alternative neurological diagnosis was identified. A clinical diagnosis of probable bvFTD was reached based on the behavioural profile, collateral history, and progression. Minimal symptomatic improvement was observed with the prescription of Quetiapine and Carbamazepine. Subsequent genetic testing identified a pathogenic C9orf72 hexanucleotide repeat expansion, confirming the diagnosis.

**Conclusion:**

This case highlights how behavioural variant frontotemporal dementia may be diagnosed clinically despite normal imaging and biomarker studies, with genetic testing serving as confirmatory tool. Careful behavioural evaluation, collateral history, and longitudinal follow-up remain essential in complex neuropsychiatric presentations. This case supports early consideration of genetic testing in diagnostically challenging cases of suspected bvFTD.

